# Case report: Clinical and genetic analysis of a family with nonsyndromic auditory neuropathy

**DOI:** 10.3389/fped.2022.1005335

**Published:** 2022-11-15

**Authors:** Lan Jiang, Hongen Xu, Danhua Liu, Sen Zhang, Ying Xu

**Affiliations:** ^1^ Department of Otorhinolaryngology Head and Neck Surgery, The Affiliated Children's Hospital of Zhengzhou University/Henan Children's Hospital/Zhengzhou Children’s Hospital, Zhengzhou, China; ^2^ Precision Medicine Center, Academy of Medical Science, Zhengzhou University, Zhengzhou, China; ^3^ The Research and Application Center of Precision Medicine, The Second Affiliated Hospital, Zhengzhou University, Zhengzhou, China

**Keywords:** auditory neuropathy, diagnosis, treatment, cochlear implant, OTOF, minigene

## Abstract

**Background:**

Auditory neuropathy (AN) is a hearing disorder caused by the failure of inner hair cells, auditory nerve synapses and/or auditory nerves. With the development of high-throughput sequencing technology, the genetic factors of AN have been revealed, and genetic testing has become an important tool for identifying different types of AN.

**Case description:**

To study the genetic cause of nonsyndromic auditory neuropathy in a Chinese family. The family was from Henan Province with three affected individuals. The audiological examinations were performed on the affected individuals, and whole-exome sequencing was carried out on the proband. The suspected pathogenic variants screened by the bioinformatic analysis were validated using Sanger sequencing in the family members. We identified three novel variants c.3277G > A (*p*.Glu1093Lys), c.4024-4G > T, and c.898-2A > G of the *OTOF* gene in the three children with AN. The first two variants were inherited from their father, and the third variant was inherited from their mother. A minigene assay was designed to test the effect of c.4024-4G > T on splicing. The variants c.3277G > A, c.4024-4G > T, and c.898-2A > G could be classified as likely pathogenic/pathogenic following the ACMG guidelines, and they are considered as the genetic causes for the patients in the family.

**Conclusion:**

New pathogenic/likely pathogenic variants of the *OTOF* gene were identified in a family with AN, enriching the mutational spectrum of the *OTOF* gene.

## Introduction

Auditory neuropathy (AN), first named by Professor Starr in 1996 (1) and also known as auditory neuropathy spectrum disorder, is an auditory dysfunction caused by the normal function of the outer hair cells, the malfunction of the inner hair cells, auditory nerve synapses and/or the auditory nerve itself (2, 3). The diagnostic criteria of AN are normal hair cell function, the eliciting of otoacoustic emission and/or cochlear microphonic potentials, abnormal auditory nerve function, and abnormal or absent auditory brainstem response (ABR) (2, 4). It is also one of the most common disorders causing hearing and speech communication impairment in infants and adolescents (4), but its exact prevalence is not known. A study by Wang in 2015 showed that approximately 0.16% of newborns had abnormal auditory nerve function, accounting for 10% of permanent hearing loss in children (5).

Determining the etiology of AN is very important to guide the choice of patients' treatment methods. At the International Newborn Hearing Screening Conference in Como, Italy, in 2008, Professor Starr suggested subdividing AN into two types: AN and auditory synaptic pathology. In the former, only the auditory nerve is involved, and the inner hair cells and synapses are normal; in the latter, the inner hair cells and/or synapses are affected, and the auditory nerve is normal (6). However, the categorization and diagnosis of AN and auditory synaptic lesions are still very difficult. In addition, patients can be subdivided into nonsyndromic and syndromic auditory neuropathies according to whether they are complicated with other systemic diseases in addition to hearing loss.

With the development of high-throughput sequencing technology, the genetic factors of AN have been gradually revealed, and genetic testing has become an increasingly important tool in the identification of different types of AN. Around 20 genes have been identified as being associated with AN, including genes associated with non-syndromic AN (OTOF, PJVK, DIAPH3, 12S rRNA, GJB2, SLC19A2, and SLC17A8), and genes associated with syndromic AN (PMP22, MPZ, NF-L, NDRG1, GJB1, GJB3, OPA1, TMEM126A, FXN, TIMM8A, WFS1, and AIFM1) (7).

The *OTOF* gene (OMIM: 603681) is located on human chromosome 2, which is approximately 100 kb long and contains 48 exons, and is the first cloned gene associated with nonsyndromic AN (8). It has been shown that the otoferlin protein encoded by the *OTOF* gene is expressed in mice's cochlear inner hair cells, which is an important component of the presynaptic structure of inner hair cells (8). The otoferlin protein is a calcium sensor that triggers the cytosolic action of neurotransmitters at the inner hair cell ribbon synapses in a calcium-dependent manner (9). Therefore, the synaptic and presynaptic types are most common in patients with *OTOF* gene mutations, and cochlear implantation has a better effect (10). As of October 2019, around 200 variants of the *OTOF* gene have been included in the Human Gene Mutation Database (HGMD). Furthermore, although most *OTOF* variants were unique to a single family, variant hotspots exist in some populations; for example, c.2485C > T is more common in the Spanish population (11).

The proportion of patients with AN clearly caused by *OTOF* variants differs from country to country and region to region. A 2016 study by Prof. Wang et al. found that *OTOF* variants were responsible for 41.2% (14/34) of hearing neuropathies in Chinese infants and children, much higher than the 5.5% (4/73) in adults, making the *OTOF* gene the main causative gene for AN in Chinese infants and children (12).

The present study examined three patients with hearing impairment from one family with a preliminary diagnosis of AN. Genetic testing was performed on the proband and candidate variants were validated in the family members. We found pathogenic/likely pathogenic variants of the *OTOF* gene were responsible for the AN in the family, enriching the *OTOF* gene's mutational spectrum.

## Case description

### Subjects

**Figure 1:**
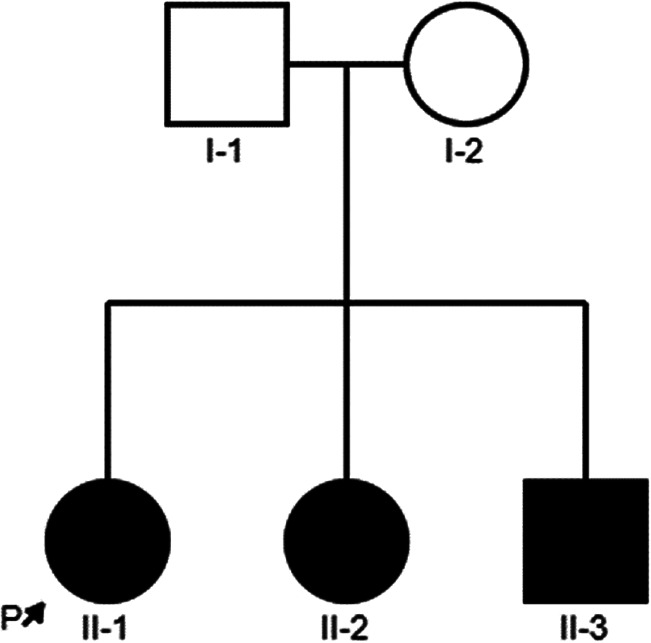
Family tree of the proband.

In this study, we described a Chinese family with nonsyndromic deafness, and three children are affected. The proband (II-1) is a 9-year-old girl, the first child in this family, and was delivered normally. She has normal growth and development but congenital deafness. Her young sister and brother were also congenitally deaf. Their parents were healthy, with normal hearing and non-consanguineous marriage. The detailed family history and medical history of the family line were investigated, and the pedigree is shown in Figure 1. Three patients (II-1, II-2, and II-3) underwent Audiological examinations,, including electric otoscopy, acoustic immittance measurement of the middle ear, distortion-product otoacoustic emission (DPOAE), ABR, and multi-frequency auditory steady-state response (ASSR). Other examinations included computed tomography (CT) and magnetic resonance imaging (MRI) of the temporal bone and skull to exclude intracranial lesions, etc.

The experimental protocol of this study was approved by the Ethics Committee of Henan Children's Hospital (approval no.: 2019-H-K16) and complied with the Declaration of Helsinki of the World Medical Association. Subjects volunteered to participate in the study. After the patients' parents signed the informed consent form, peripheral blood was drawn from the three patients and their parents for genetic testing.

### Methods

#### Exome sequencing

Genomic DNAs were extracted from peripheral blood samples of five family members according to the manual of the Whole Blood Genomic DNA Extraction Kit (Thermo Scientific, United States). DNA concentration and purity were measured using NanoDrop One (Thermo Scientific, United States). The DNA sample was fragmented into 250 bp using an ultrasonic interrupter (Bioruptor® Pico Sonication System, Diagenode, United States); quality control of the fragmented DNA was performed using the Agilent 4200 TapeStation System (Agilent Technologies Inc., United States). The procedure for the Agilent SureSelect Human All Exon V6 (Agilent Technologies Inc., United States) for library construction and gene exon capture was then followed. The resulting library was sequenced using the Illumina HiSeq 4000 platform (Illumina, United States) with pair-ends 150 bp.

#### Bioinformatics analysis and variant interpretation

The sequenced reads were mapped against the human reference genome (NCBI37/hg19) using Burrows-Wheeler Aligner (BWA) package (13). Analysis of single nucleotide polymorphisms (SNPs) and Indels (inserts and deletions) were using GATK (Genomic Analysis Tool Kit) HaplotypeCaller v. 4.1.2 (14). The variants were annotated using variant effect predictor software (15) with a number of annotation databases including ClinVar (16), HGMD (17), 1,000 genome Project (18), the Exome Aggregation Consortium database (ExAC) (19), the Genome Aggregation Database (gnomAD) (20), and a database for predicting the function of missense variants (21). The selected candidate variants were interpreted for pathogenicity according to the American College of Medical Genetics and Genomics (ACMG) (22) and Expert specification of the ACMG/AMP variant interpretation guidelines for genetic hearing loss (23).

#### Sanger sequencing

The variants identified by the next-generation sequencing assays were validated in all participating members using polymerase chain reaction (PCR) and Sanger sequencing. Primers were designed for each of the three variants using NCBI Primer-BLAST software (see Table 1) and synthesized by Shanghai Shangya Biotechnology Co., Ltd. (China). The PCR reactions were performed using Taq 2× Master Mix kits (Shanghai Novoprotein Technology Co., Ltd., China), and the amplified products were identified using 2.2% agarose gel electrophoresis for fragment size, before being purified using DNA product purification kits (Shanghai Lifefeng Biotechnology Co., Ltd., China). The purified PCR products then underwent Sanger sequencing using an Applied Biosystems sequencer (Applied Biosystems, United States).

**Table 1:** Primer sequences.

Primer name	Sequence of primer (5″–3′)
OTOF-F1	ACCCAAGCTAGGTGTGATATTTA
OTOF-R1	GGTCTGCTCCTAGGCTCTCACCTT
OTOF-F2	AGAGGCTGCCTGTTGTCGTCTCTA
OTOF-R2	GAAGACTGTCTTCATGCCCTTTG
MINI-OTOF- BamH1-F	GCTCGGATCCTGTGTCCAAGTGTTCTCATTGTTC
MINI-OTOF-EcoR1-R	TGCAGAATTCAAAACTAATTTGTTCCTTCTAGAAC

#### Minigene experiment

We performed a functional analysis of the c.4024-4G > T variant with an *in vitro* minigene assay to verify its impact on splicing. Minigenes were constructed by inserting exon 33 and part of the flanking introns into the pcMINI vector, which contained the universal ExonA-IntronA-MSC-IntronB-ExonB. The cells were transfected and then observed for abnormalities in the splicing pattern of ExonA-Exon33-ExonB. Primers were designed for three rounds of PCR. In the first PCR experiment, genomic DNA was amplified using specific primers OTOF-F1 and OTOF-R1. The second PCR used primers OTOF-F2 and OTOF-R2 to amplify the products of the first round of PCR. The third round of PCR was performed using the products of the second PCR as templates and MINI-OTOF-BamH1-F and MINI-OTOF-EcoR1-R as primers to obtain wild-type and mutant fragments of the *OTOF* gene (the fragment contained the intron 32, exon33, and intron 33 parts of the *OTOF* gene, and the primer sequences are shown in Table 1). The final round of PCR product was purified, and the wild-type and mutant fragments were ligated with pcMINI vectors to obtain two recombinant vectors, pcMINI-wt and pcMINI-mut. The recombinant vectors were transfected into 293 T and HeLa cell transiently according to the liposome instructions. After 36 h, total RNA was extracted from each plate of cells and RT-PCR was performed. After detecting the band size using agarose electrophoresis, sequencing was performed, and abnormalities in the splicing pattern were determined.

## Results

### Clinical hearing characteristics

The three patients (II-1, II-2, and II-3) showed no significant abnormalities in the bilateral tympanic membranes on electric otoscopy and no significant abnormalities in the bilateral external auditory canal walls, tympanic membranes, tympanic chambers, auditory ossicles, and internal auditory canals on CT plain scans of the temporal bone; no significant abnormalities were detected on MRI either. The proband (II-1) passed a DPOAE test for both ears, and an ABR test revealed response thresholds >100 dB in both ears. She was therefore diagnosed with extremely severe deafness in both ears. A multifrequency ASSR test also revealed extremely severe sensorineural hearing loss (SNHL) in both ears (500-1K-2K-4KHz, left ear: 90-110-100-90dBnHL; right ear: 90-non-responsive-110-non-responsive), which is consistent with the audiological features associated with AN. In the absence of any systemic disease other than hearing loss, and in combination with their clinical manifestations and various examination findings, all three patients were diagnosed with nonsyndromic AN.

### whole-exome sequencing

Whole-exome sequencing was performed on the proband, and 13 G of raw data was obtained. The proportions of base quality values ≥30 (Q30) were all greater than 90%, and. the coverage of target regions was greater than 99.5%. The average sequencing depth for the target region was 130–160×, with more than 98% of the target sequence reaching up to 20×.

**Figure 2:**
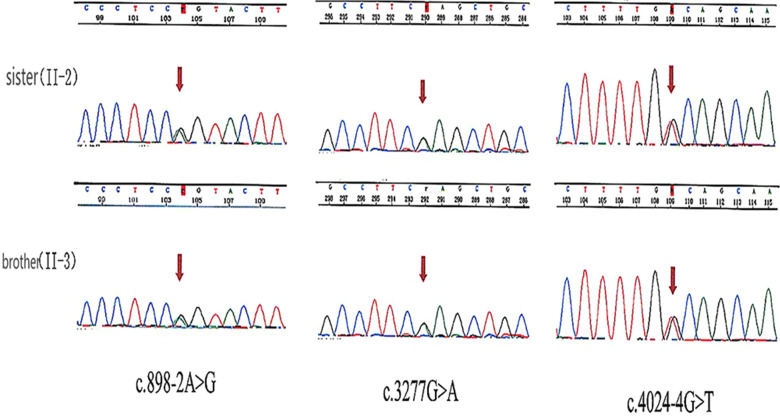
Sanger sequencing results.

Bioinformatic analysis has identified three variants (NM_194248.2) c.898-2A > G, c.3277G > A, and c.4024-4G > T in the *OTOF* gene. The variants c.3277G > A and c.4024-4G > T were inherited from the father and c.898-2A > G was inherited from the mother (see Table 2). Sanger sequencing revealed that both the sister and brother of the proband (II-2 and II-3) carried three variants, thus confirming the co-segregation of the three *OTOF* variants with the phenotype in the family (see Figure 2).

**Table 2:** Gene assay results.

Gene	Transcript	Nucleotide alterations	Amino acid alterations	Zygote type	Mutation Source	Mutation rating	ACMG Evidence
*OTOF*	NM_194248.2	c.898-2A > G	/	Heterozygous	Mother	Pathogenic	PVS1,PM2_Supporting,PP1_Moderate
*OTOF*	NM_194248.2	c.3277G > A	*p*.Glu1093Lys	Heterozygous	Father	Likely pathogenic	PM2_Supporting,PM3,PP1_Moderate,PP3
*OTOF*	NM_194248.2	c.4024-4G > T	/	Heterozygous	Father	Likely pathogenic	PS3_Moderate,PM2_Supporting,PM3,PP1_Moderate

### Minigene experiment

**Figure 3:**
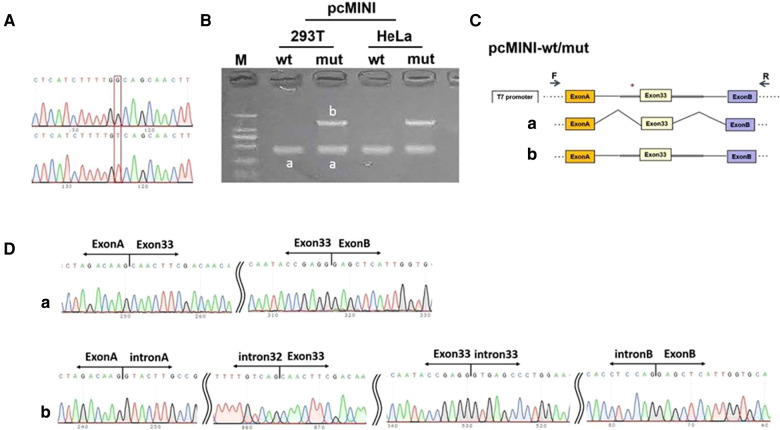
Minigene experiment. (**A**) A sequencing map was constructed using minigenes, with wt at the top and mut at the bottom. (**B**) Real-time polymerase chain reaction transcription analysis of the agarose gel electrophoresis diagram was performed, and the differential bands in the 293T and HeLa cells were marked as A and B, respectively. (**C**) Schematic diagram of the minigene construction and splicing. (**D**) Diagram of sequencing results corresponding to the spliced bands. Red * indicates the mutation location.

A real-time PCR (RT-PCR) assay showed that the wild type (OTOF-wt) in HeLa and 293 T cells had only one band (named band A) of normal size (see Figure 3), the mutant type (OTOF-mut) had two bands, with the small band being similar in size to band A and the large band being named band B (see Figure 3). Both bands were sequenced, and the results showed that band A was normal splicing, with the splicing pattern being ExonA-Exon33 (67 bp)-ExonB, and band B was abnormal splicing that retained all intact introns in the insertion vector; its splicing pattern was ExonA-IntronA-Intron32 (709 bp)-Exon33 (67 bp)-Intron33 (345 bp)-IntronB-ExonB. There was more pronounced intron retention in the mutant type, although a band A consistent with the wild-type splice pattern was present; in the wild-type minigene there was no band B of intron retention at all, so the results of the minigene experiment suggested that variant c.4024-4G > T causes ∼50% of abnormal splicing.

### Variant interpretation

The variant c.4024-4G > T was inherited from the patient's father and was not found in the database of normal population in the 1000 Genomes Project and gnomAD, allowing the use of PM2_supporting evidence. The *in vitro* minigene experiment indicated that c.4024-4G > T variant affects splicing, allowing the use of PS3_Moderate evidence. A pathogenic mutation was detected in trans of this variant in the family, allowing the use of PM3 evidence. Sanger sequencing confirmed the variant was co-segregated with the phenotype in the family, allowing the use of PP1_Moderate evidence. The c.4024-4G > T variant could be classified as likely pathogenic according to the ACMG guidelines for the interpretation of genetic variants (22).

The missense variant c.3277G > A was also inherited from the patient's father and had a very low frequency in the database of normal population in the 1000 Genomes Project and gnomAD, allowing the use of PM2_supporting evidence. A pathogenic mutation was detected in trans of this missense variant in the family verified by parents, allowing the use of PM3 evidence. All three patients with AN in the family carried this variant, so the variant was co-segregated with the phenotype in the family, allowing the use of PP1_Moderate evidence. Multiple lines of computational evidence support a deleterious effect on the gene or gene product, allowing the use of PP3 evidence. The c.3277G > A variant could be classified as likely pathogenic according to the ACMG guidelines (22).

The variant c.898-2A > T was inherited from the patient's mother. The variant frequency in the 1000 Genomes Project and gnomAD was 0, allowing the use of PM2_supporting evidence. The variant had definitive gene-disease validity in ClinGen and was a null variant (canonical -2 splice sites) in the *OTOF* gene, where LOF is a known mechanism of disease, allowing the use of PVS1 evidence. The variant was co-segregated with the phenotype in the family, allowing the use of PP1_Moderate evidence. The c.898-2A > G variant could be classified as pathogenic according to the ACMG guidelines (22).

## Discussion

In this study, three heterozygous variants of the *OTOF* gene were detected in all three affected individuals, with variants c.3277G > A (*p*.Glu1093Lys) and c.4024-4G > T coming from the father and variant c.898-2A > G from the mother. The parents were all heterozygous carriers without hearing impairment. The variant c.898-2A > G from the mother is a canonical -2 splicing variant. The other two variants were found in cis in the present study and a previous study (24), and it suggests that they probably constitute a pathogenic haplotype. In the study by Thorpe et al., the authors have considered both variants as causal ones. To determine the effect of c.4024-4G > T on splicing, we constructed minigene vectorsand performed RT-PCR at a cellular level. The results showed that variant c.4024-4G > T caused ∼50% of abnormal splicing. The three variants were co-segregated with hearing loss phenotype in the family members and they were interpreted according to the ACMG criteria and the guidelines for the classification of genetic variants, with c.898-2A > G as pathogenic and c.3277G > A as well as c.4024-4G > T as likely pathogenic. This indicated that the three variants were likely to be involved in the pathogenesis of the disorder in this family.

AN is an auditory disorder caused by genetic abnormalities that affect speech comprehension in addition to hearing and is an important type of disease in hereditary deafness. There are many genes that can cause the disease, of which *OTOF* is one of the most important (25). A number of studies have been conducted to explore the function of the *OTOF* gene, and a total of 455 classified variants of this gene have been reported in UniProt and ClinVar, of which 64 variants are classified as pathogenic/likely pathogenic and 212 are classified as of unknown clinical significance. Of the 64 pathogenic/possibly pathogenic variants, approximately 42% are missense mutations, approximately 20% are nonsense mutations, and approximately 19% are frameshift mutations. AN is a heterogeneous disease caused by the *OTOF* gene, it, and the outcome of cochlear implantation varies among patients (26). Some studies have reported that the outcome of cochlear implantation in patients with specific genetic mutations is predictable (27), so genetic diagnosis is of great importance for clinical management.

One study performed a genetic sequencing analysis on a large number of deafness-causing genetic variants in order to determine the frequency of associated hearing loss and the precise genetic and clinical background. Iwasa et al. performed genetic testing on 2,265 patients who had SNHL consistent with autosomal recessive inheritance from 53 otolaryngology departments in Japan. Of these, 39 patients carried homozygous or compound heterozygous variants in the *OTOF* gene, accounting for approximately 1.72% of cases. Among them, 32 patients had permanent hearing loss, seven had severe hearing loss, and one had mild hearing loss. The patients with biallelic sites of *OTOF* gene variants had severe or permanent hearing loss (28). The study has also demonstrated that patients with *OTOF* pathogenic variants would be good candidates for cochlear implantation; therefore, detecting *OTOF* pathogenic is quite beneficial for patients with ANSD.

## Conclusion

In summary, we identified three heterozygous variants in the *OTOF* gene, c.898-2A > G, c.3277G > A, and c.4024-4G > T, as the genetic causes of three patients of AN using next-generation sequencing and Sanger sequencing. The last two variants were found in cis in the present study and a previous study, and they probably constitute a pathogenic haplotype. There is sufficient evidence to classify the c.3277G > A as a likely pathogenic variant conforming with the ACMG Guideline. Minigene experimental techniques showed that the intron variant c.4024-4G > T affects ∼50% of splicing and leads to intron retention, which may further contribute to the pathogenicity. The discovery of new variants enriches the mutational spectrum of the *OTOF* gene and provides a new reference for the genetic diagnosis of AN.

## Data Availability

The original contributions presented in the study are included in the article/Supplementary Material, further inquiries can be directed to the corresponding author/s.
